# Motoric Cognitive Risk Syndrome: Prevalence and Cognitive Performance

**DOI:** 10.1093/geroni/igab046.2277

**Published:** 2021-12-17

**Authors:** Elkin Garcia-Cifuentes, Isabela Marquez, Carlos Cano

**Affiliations:** Pontificia Universidad Javeriana, Pontificia Universidad Javeriana, Distrito Capital de Bogota, Colombia

## Abstract

Cognitive decline and dementia have a significant impact older adult. Motor Cognitive Risk Syndrome (MCRS) is a pre-dementia stage where slow gait speed and subjective memory complaints are present. MCRS increases the risk of frailty, dementia, disability, falls and overall mortality. We used data from the SABE Colombia study (Health, Well-Being, and Aging) conducted in 2015 in adults aged 60 years and older. After adjusting for confounding variables MCRS was associated with MMSE (OR 0.90, CI 0.82-0.99), pre-frail (OR 9.1, CI 3.26-25.47) and frail (OR 21.38, CI 6.30-72.57). This study found a prevalence of 5.45% of MCRS in Colombian older adults. We found an associations between cognitive performance (MMSE), frailty and MCRS. Our results increase the awareness of a pre-dementia stages different to Mild Cognitive Impairment (MCI), as these individuals are at greater risk than those with MCI to develop dementia.

